# Supplementary material for: Synaptic Zn2+ potentiates the effects of cocaine on striatal dopamine neurotransmission and behavior
Source: Transl Psychiatry. 2021 Nov 8;11:570. doi: 10.1038/s41398-021-01693-0 (PMC8575899; doi:10.1038/s41398-021-01693-0)
Supplement: Supplementary file 1 — Supplemental Information [file 41398_2021_1693_MOESM1_ESM.docx]

**Fig. S1.** **Elemental profiling in postmortem striatal tissue from cocaine users (COC) (n=19) and control (CTL) (n=20) subjects using total reflection X-ray fluorescence spectroscopy. Ppm – parts per million. Data expressed as Mean ±SEM.**


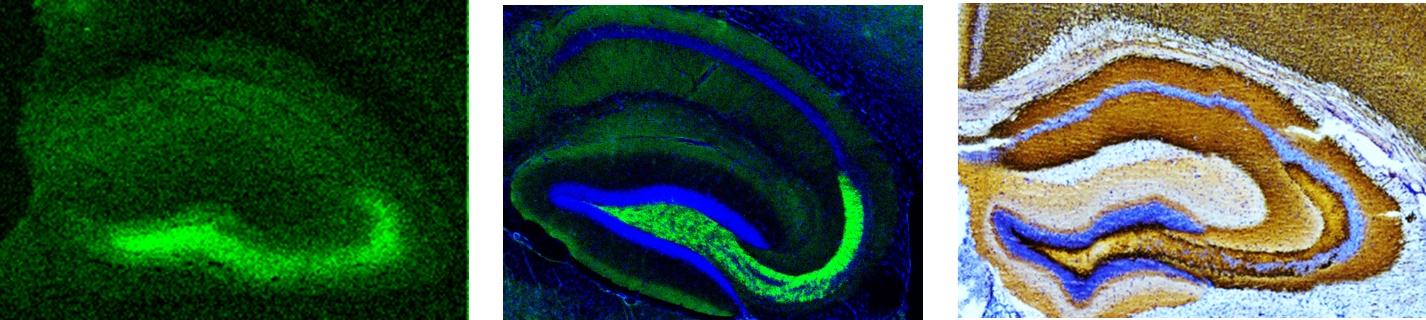


**Fig. S2. Visualization of synaptic Zn^2+^ using synchrotron X-ray fluorescence microspectroscopy (XRFS). XRFS (left) signal overlaps with ZnT3 immunohistochemistry (middle), and histochemically-reactive synaptic Zn^2+^ stain (right) in hippocampus.**

**
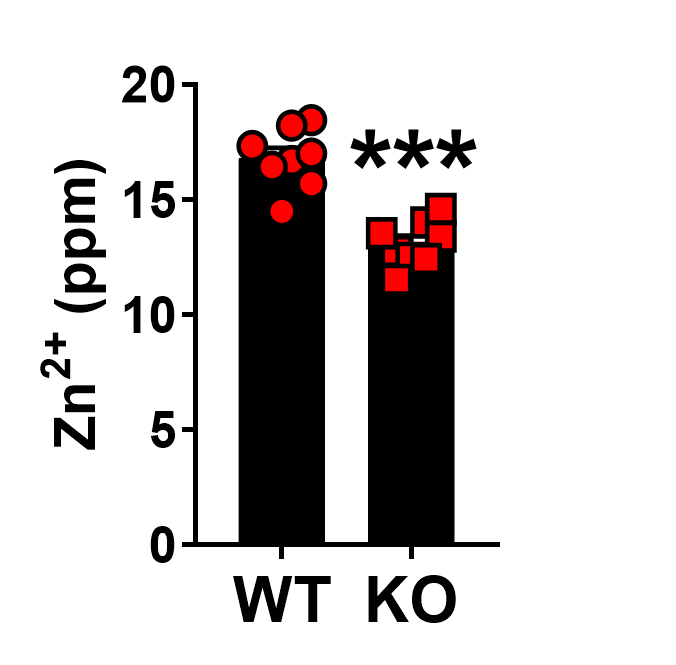
**

**Fig. S3. ZnT3 knockout (KO) mice have significantly lower (unpaired t-test; t=6.419; p<0.001) Zn^2+^ in cortex compared to wildtype (WT) mice as assessed using total reflection X-ray spectroscopy (TXRF). ***p≤0.001**. **Data expressed as Mean ±SEM**.


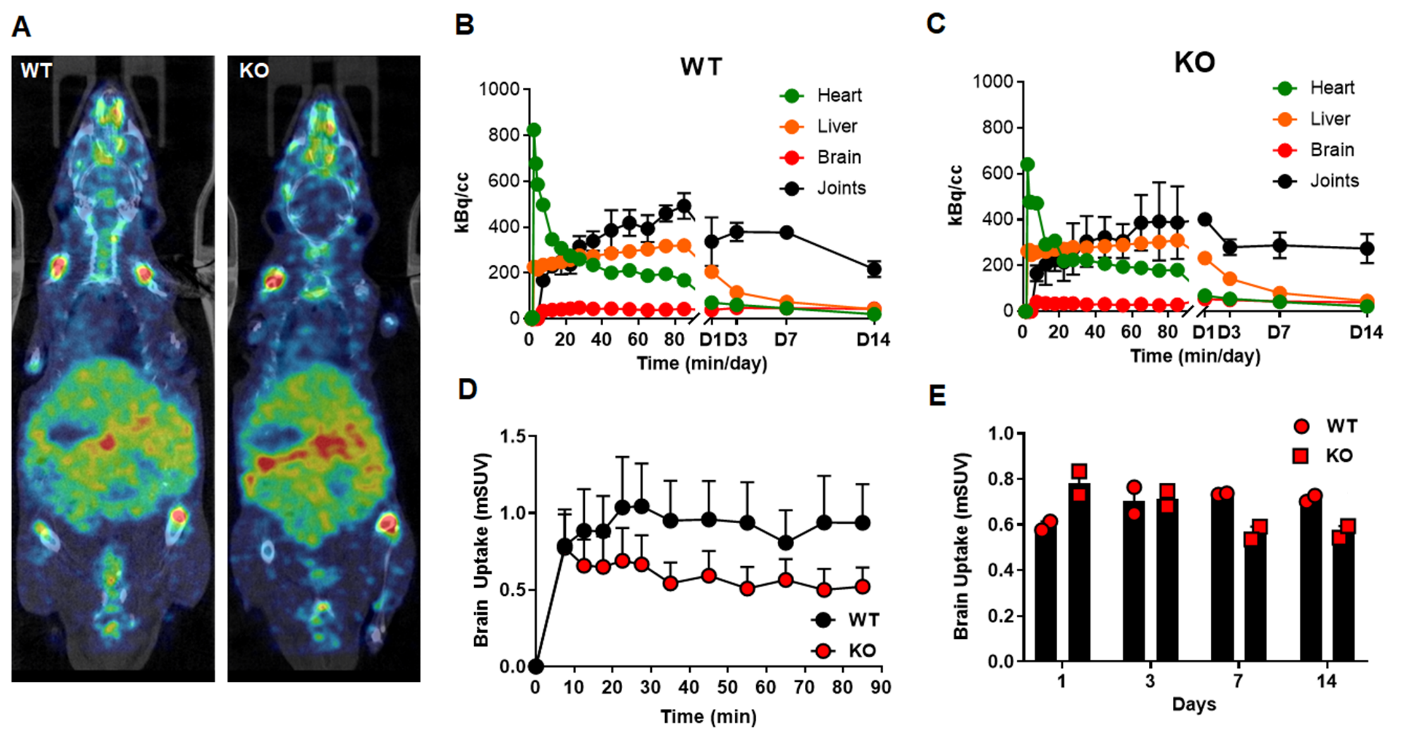


**Fig. S4. (A) Representative whole-body PET images (horizontal plane) of ^65^Zn uptake in a wildtype (WT) and a ZnT3 knockout (KO) mouse. (B) ^65^Zn time activity curves in different organs or body regions in WT and (C) ZnT4 KO mice. (D) ^65^Zn brain uptake over the first 90 min after ^65^ZnCl_2_ intravenous injection. (E) ^65^Zn brain uptake at different days after ^65^ZnCl_2_ intravenous injection showing that KO mice differed in brain uptake at 1, 7, and 14 days. Data expressed as Mean ±SEM.**


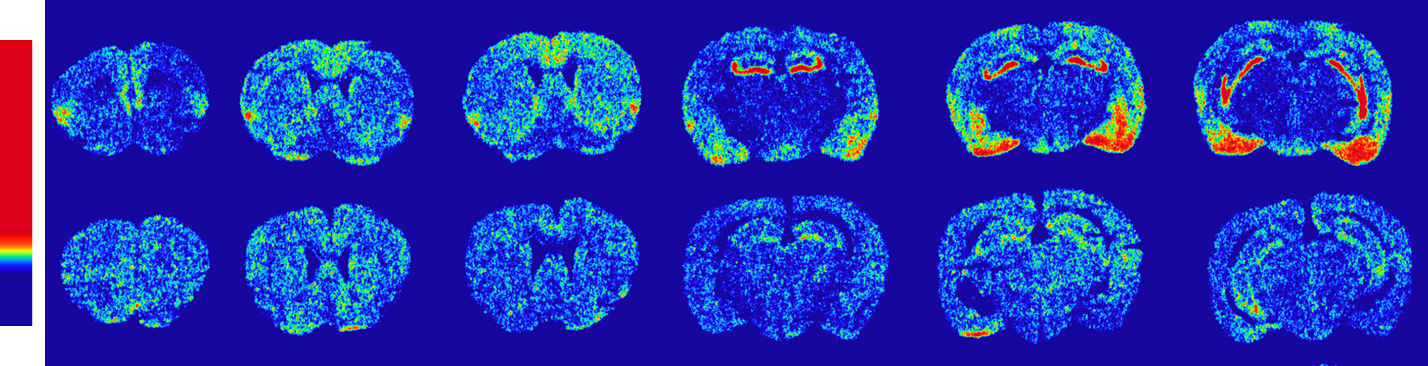


**Fig. S5. *Ex vivo* autoradiography at 15 days after intravenous ^65^Zn injection in a wildtype (top row) and a ZnT3 knockout mouse (bottom row).**


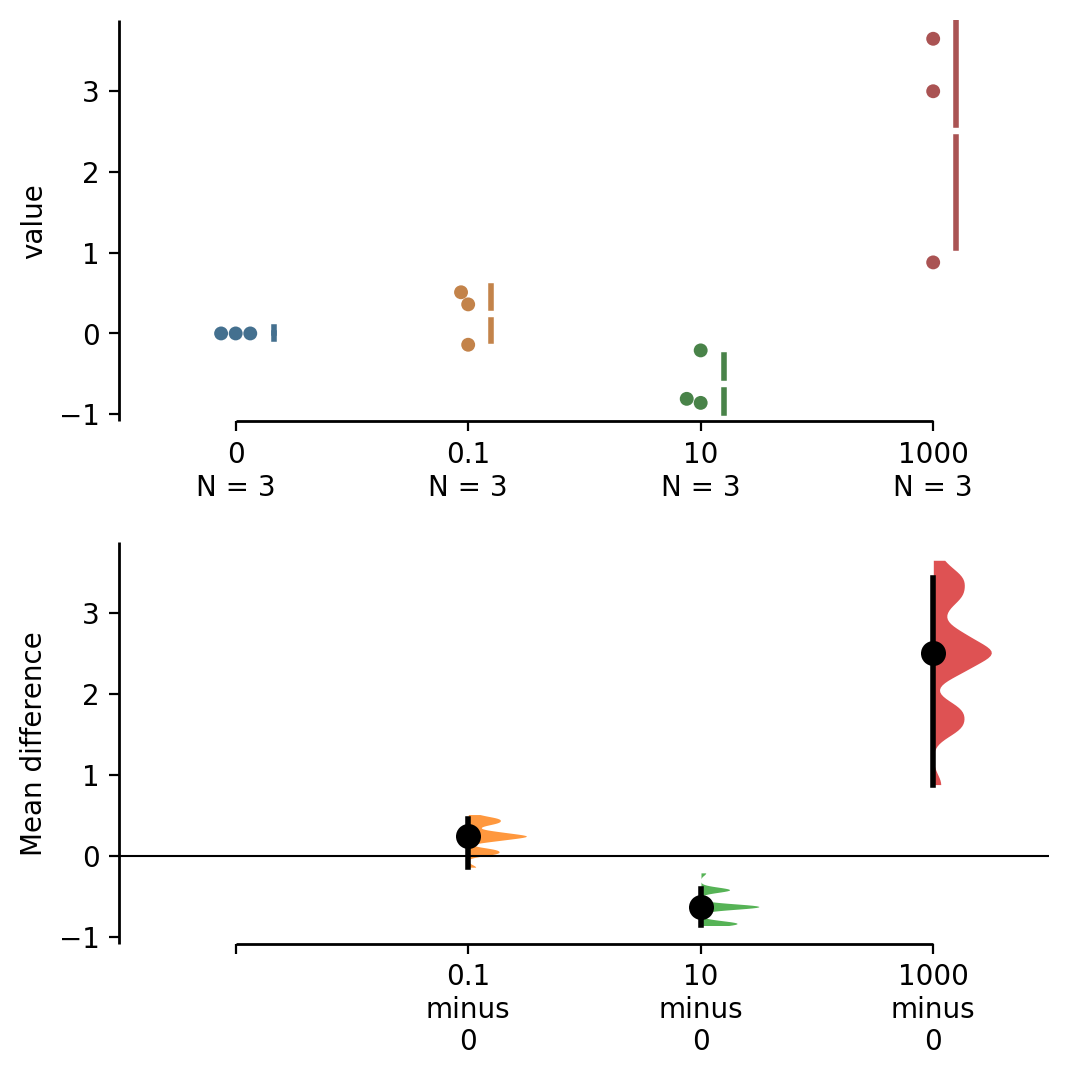


**Fig. S6. Cumming estimation plot showing the mean difference for the 3 comparisons of Zn^2+^ concentrations and their effects on [^3^H]WIN35,428 binding affinity (0 vs. 0.1 µM, 0 vs. 10 µM, 0 vs 1000 µM).**

|  | **WT** | **KO** | **ANOVA** |
| --- | --- | --- | --- |
| **DA_Max_** | 0.042 ± 0.007 μM (n=4) | 0.098 ± 0.035 μM (n=4) | F_1,6_ = 2.4, p = 0.172 |
| **Clearance (k)** | 2.8 ± 0.6 μM/s (n=4) | 1.5 ± 0.4 μM/s (n=4) | F_1,6_ = 3.8, p = 0.099 |

**
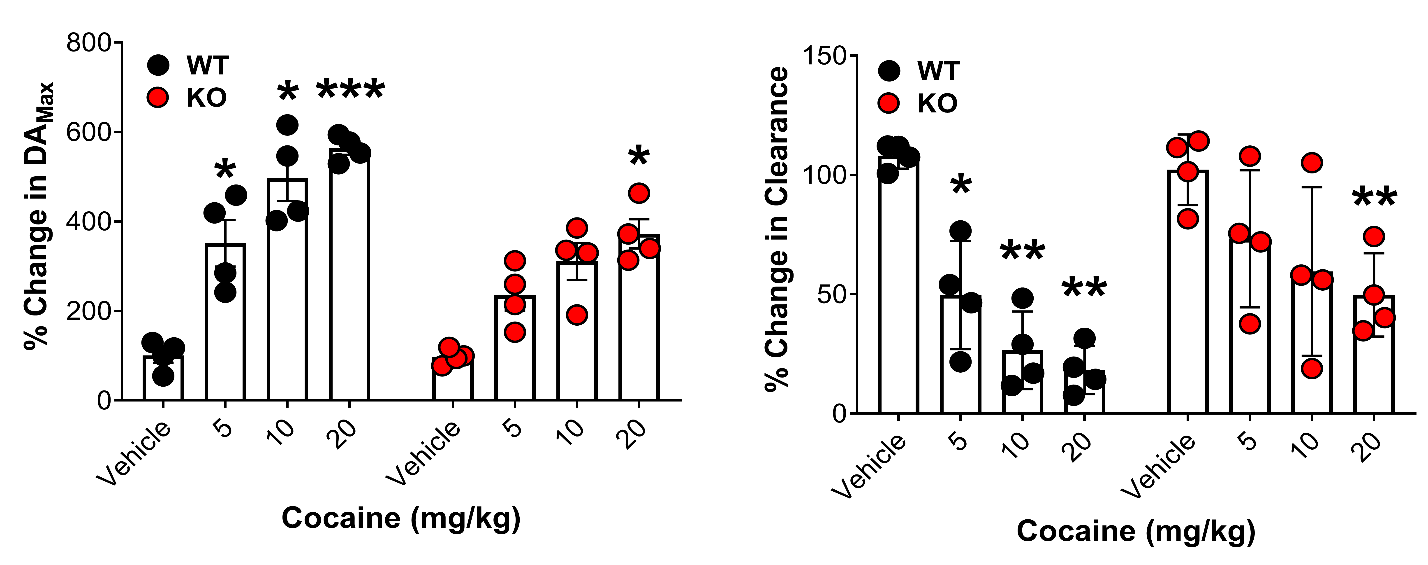
**

**Fig. S7. Upper: Table showing baseline fast scan cyclic voltammetry measures and statistics of DA_MAX_ and Clearance (k) in wild-type (WT) and ZnT3 knockout (KO) mice. Lower: Data from each 5 min bin from Figure 3 were averaged per treatment (e.g., vehicle, 5, 10 and 20 mg/kg). *DA_MAX_:* A 2-way repeated measures (RM) ANOVA (significant interaction effect: F(3, 18)=6.42; p=0.003) showed that WT mice had significantly greater DA_MAX_ (Holm-Sidak multiple comparisons) at 5 mg/kg (t=5.39; p=0.03), 10 mg/kg (t=8.18; p=0.01) and 20 mg/kg (t=31.1; p<0.001) cocaine as compared to Vehicle, whereas ZnT3 KO mice had significantly greater DA_MAX_ (Holm-Sidak multiple comparisons) only at 20 mg/kg (t=7.63; p=0.02) cocaine as compared to Vehicle. *Clearance:* A 2-way RM ANOVA (significant interaction effect: F(3, 18)=5.44; p=0.007) showed that WT mice had significantly greater Clearance (Holm-Sidak multiple comparisons) at 5 mg/kg (t=6.53; p=0.02), 10 mg/kg (t=10.75; p=0.008) and 20 mg/kg (t=17.44; p=0.002) cocaine as compared to Vehicle, whereas ZnT3 KO mice had significantly greater Clearance (Holm-Sidak multiple comparisons) only at 20 mg/kg (t=9.73; p=0.01) cocaine as compared to Vehicle. *p<0.05, **p<0.01, ***p<0.001. All data shown as Mean ±SEM.**


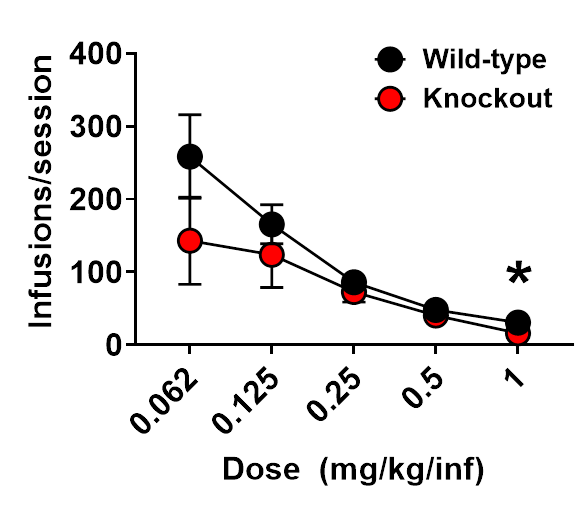


**Fig. S8. Infusions per session across different doses of intravenous cocaine self-administration between wild-type and ZnT3 knockout mice (t=2.54; p=0.03 at 1 mg/kg/infusion). Data expressed as Mean ±SEM.**
